# Associations between cognitive performance and sigma power during sleep in children with attention-deficit/hyperactivity disorder, healthy children, and healthy adults

**DOI:** 10.1371/journal.pone.0224166

**Published:** 2019-10-24

**Authors:** Arnika Bestmann, Annette Conzelmann, Lioba Baving, Alexander Prehn-Kristensen

**Affiliations:** 1 Department of Child and Adolescent Psychiatry and Psychotherapy, Centre for Integrative Psychiatry, University Hospital Schleswig-Holstein, Kiel, Germany; 2 Department of Child and Adolescent Psychiatry, Psychosomatics and Psychotherapy, University of Tübingen, Tübingen, Germany; 3 PFH—Private University of Applied Sciences, Department of Psychology (Clinical Psychology II), Göttingen, Germany; Carl von Ossietzky Universitat Oldenburg, GERMANY

## Abstract

Sigma power during sleep is associated with cognitive abilities in healthy humans. We examined the relationship between sigma power in sleep EEG and intelligence and alertness in schoolchildren with ADHD (n = 17) in comparison to mentally healthy children (n = 16) and adults (n = 23). We observed a positive correlation between sigma power in sleep stage 2 and IQ in healthy adults but a negative correlation in children with ADHD. Furthermore, children with ADHD showed slower reaction times in alertness testing than both control groups. In contrast, only healthy children displayed a positive correlation between sigma power and reaction times. These data suggest that the associations between sigma power and cognitive performance underlie distinct developmental processes. A negative association between IQ and sigma power indicates a disturbed function of sleep in cognitive functions in ADHD, whereas the function of sleep appears to be matured early in case of motor-related alertness performance.

## Introduction

Sleep is essential for optimal cognitive performance such as alertness, working memory, or IQ performance [1–4]. Sleep is usually understood as being a state that can easily be disturbed, and sleep quality can change from one day to the other depending on many different factors [5]. Although sleep quality can significantly influence cognitive performance in children and adults, only little is known about a state-independent relationship between sleep and cognitive functions in terms of an individual trait. Meanwhile, there is increasing evidence that sleep spindle or sigma activity can predict state-independent cognitive functions such as IQ performances in healthy adults (e.g. [6, 7, 8]). Studies on the relationship between sigma activity and cognitive performance with respect to brain maturation, however, are clearly underrepresented.

Not only cognitive performance increases over the course of maturation but sleep also changes dramatically from childhood to adulthood: While deep sleep stages 3 and 4 as well as rapid-eve movement (REM) sleep decrease, the amount of sleep stage 2 (S2) increases [9, 10]. Hallmarks of S2 are the occurrence of sleep spindles which are short bursts of EEG activity mainly in the sigma frequency range of 12-16Hz. Sleep spindle activity and sigma power changes over the course of maturation, as well: There is an increase in spindle density in children up until the age of 11 which tends to decline in adolescence [11]. In the same way, sigma power increases within the first decade of life and declines until adulthood [12, 13].

To date, there have been some studies in children and adolescents emphasizing a relationship between cognitive functions and sleep spindle or sigma activity (e.g. [14, 15], for review see [16]). A current meta-analysis including 12 studies in adolescents concluded that sleep spindles are related to cognitive performance (such as non-verbal IQ, working memory, and motor skilled accuracy) [17]. One recent study emphasizes that the development of sleep spindles across adolescence is related to an age-related increase in IQ performance [18]. Therefore, it is plausible to assume that the maturation of sigma brain activity during sleep is an indicator of the increase in cognitive abilities in healthy individuals.

Attention-deficit/hyperactivity disorder (ADHD) is a neurodevelopmental disorder characterized by the core symptoms of inattention, hyperactivity, and impulsivity [19] affecting 2.6–4.5 percent of children worldwide [20]. Children with ADHD display deficits in several cognitive domains ranging from simple alertness performance to intellectual capacities [21–24]. Besides this, ADHD is accompanied by subjective sleep complaints [25–28]. However, meta-analyses on objective data have yielded mixed findings [27, 29, 30]. There is one study which reported decreased sigma activity during sleep stage 2 [31]. Another study reported that sleep spindles are accompanied by reduced oscillatory brain activity before and after the spindle in children with ADHD [32]. In our previous studies, we did not observe such quantitative differences in sleep architecture. However, we found a lack of associations between slow oscillatory brain activity during sleep and declarative memory performance [33, 34]. Therefore, we concluded that brain oscillations during sleep are less effective in supporting cognitive functions in ADHD than in healthy children.

The present study investigated associations between sigma power during sleep and cognitive performance in healthy and abnormal neurodevelopment. Here, we refer to some data that has been previously published elsewhere in part [34]. The aim of the study is to show that sigma activity during sleep can serve as an indicator for cognitive abilities in both healthy children and healthy adults. ADHD is a neurodevelopmental disorder, and children with ADHD were shown to display reduced functions of oscillatory brain activity during sleep with respect to memory performance. Therefore, we assume likewise that the association between sigma activity during sleep and cognitive ability is affected in those patients, as well. Taken together, we expect that sigma power is only correlated with intelligence and alertness performance in healthy children and adults but not in children with ADHD.

## Methods

### Participants

Participants were 17 boys with ADHD [35] aged 9–12 years, 16 healthy male children (HC) aged 9–12 years, and 23 healthy adults (HA) aged 20–31 years (see also Table 1). All participants were Caucasians. Children were recruited from our psychiatric outpatient department and by announcements in a local newspaper. Adult participants (mainly students from Kiel University) were recruited by announcements. In most parts the study, the design and study sample were consistent with one of our previous publications [34]. For the current analyses, four more participants (one ADHD patient, three adults) were included. Children with and without ADHD did not differ with respect to age [t(31) = 0.63, p = .531]. All participating adults, children and their parents gave written, informed consent after the procedures had been fully explained. Participants were reimbursed for their participation. The study was approved by the local ethics committee of the medical faculty of Kiel.

**Table 1:** Participants´ characteristics and results.

	ADHD (n = 17)	HC (n = 16)	HA (n = 23)	ANOVA		t-test		
						ADHD vs. HC	ADHD vs. HA	HC vs. HA
	mean (SD)	mean (SD)	mean (SD)	F	p	p	p	p
Age	10.7 (1.05)	10.9 (1.05)	24.95 (2.86)	335.2	**< .001**	.531	**< .001**	**< .001**
IQ	106.4 (13.8)	107.9 (9.0)	113.2 (11.5)	1.92	.156	-	-	-
RT (ms)	328.2 (65.4)	288 (30.6)	232 (14.0)	28.7	**< .001**	**.031**	**< .001**	**< .001**
TIB (min)	601 (64.7)	580 (59.3)	492 (64.0)	17.0	**< .001**	.368	**< .001**	**< .001**
TST (min)	520 (58.1)	513 (58.6)	431 (58.4)	14.4	**< .001**	.731	**< .001**	**< .001**
SE	87 (8.4)	88.5 (7.2)	88.1 (7.1)	0.2	.852	-	-	-
S1 (%)	5.4 (3.3)	5.2 (1.6)	8.3 (3.5)	6.6	**.003**	.835	.011	**.003**
S2 (%)	44.2 (6.9)	48.2 (11.5)	51.1 (7.5)	3.1	.052	-	**-**	-
S3 (%)	12.4 (3.1)	13.5 (5.7)	11.1 (4.1)	1.4	.245	-	-	-
S4 (%)	18.5 (3.5)	17.2 (3.6)	8.3 (5.2)	33.2	**< .001**	.284	**< .001**	**< .001**
REM (%)	19.5 (4.5)	20.1 (4.7)	21.2 (4.3)	0.8	.462	-	-	-

Note: ADHD, attention-deficit hyperactivity disorder; HC, healthy children; HA, healthy adults; RT, reaction times; sleep parameters: TIB, time in bed; TST, total sleep time; SE, sleep efficiency; S1-4, sleep stages 1–4; REM, rapid-eye-movement; SD, standard deviation; ANOVA, analysis of variance; bold values indicate survival of a Bonferroni-corrected significance level of p < .0083 (0.05/6) chosen for all sleep parameters; see also S3 Table for statistical values (including t-values and CL-95%) of all group-wise comparisons of single means.

Children and their parents separately underwent a diagnostic interview using the German translation of the Revised Schedule for Affective Disorders and Schizophrenia for School-Age Children (Kiddie-SADS-PL [36, 37]) to assess psychiatric symptoms. Clinical interviews were conducted by experienced psychologists. According to this interview, eight ADHD patients displayed an inattentive subtype, two a hyperactive/impulsive subtype, and seven a combined subtype (for detailed information see S1 Table). We screened adult participants for possible psychiatric symptoms by using the German version of the Structured Clinical Interview for DSM-IV (SKID I/II [38] and a short German version of the symptom checklist (SCL-90-R; cut-off t-value > 60 [39]). In addition, adults worked on the German ADHD self-rating scale (ADHS-SB; cut-off > 15; [40]) and the short version of the Wender-Utah-Rating-Scale (WURS-k; cut-off > 30 [41]) to ensure that none of the adults displayed serious ADHD symptoms at the time of the study nor during childhood. Five ADHD patients were free of medication; 12 patients received methylphenidate (MPH) but discontinued taking medicine for at least 48 hours before the experimental sessions (approx. 12 half-lives).

Exclusion criterion was any comorbidity besides ADHD and oppositional defiant disorder (ODD, 6 patients additionally affected) in the ADHD group and any psychiatric disorders in the healthy children and adult group. Adult participants did not report sleep-related disorders in the Pittsburgh Sleep Quality Index (PSQI; cut-off > 5 [42]). For both children’s groups, a self-constructed sleep questionnaire was used which had already been employed in other studies before [43, 44]. This questionnaire was inspired by the PSQI as well as by a pediatric sleep questionnaire provided by the German Society of Sleep Medicine and Research (DGSM). Here, parents were asked to rate their child’s current and general health status (e.g. infections, vaccinations, medication, asthma, obesity, enlarged tonsils), specific sleep complaints (e.g. daytime tiredness, awaking at night, bed time resistance), sleep schedule (e.g. regular times of sleep, naps), and sleep-related breathing problems (e.g. irregularly breathing, snoring, sleep apnea). An English translation of this questionnaire can be found in the supplemental material (S1 File “Sleep questionnaire”). According to this questionnaire none of the children suffered from any sleeping disorder. Due to weak EEG data quality, four participants were not included in any of the analysis (not included in participant description).

### Neuropsychological performance

To screen for general cognitive abilities, Part 1 of the Culture Fair Intelligence Test (CFT-20-R [45]) was applied during the screening session. The CFT-20-R is a speed-limited, non-verbal intelligence test containing four subtests of “completing a sequence”,”classification”,”matrices”, and”topological conclusions”. The test is normed for children and adults of ages 8.5–19 and 20–60 years and is characterized by high levels of validity, reliability, and objectiveness [45]. The CFT-20-R is separated into two parts, both parts being summed up for one general factor value. We used Part 1 only (approx. 25 min in duration), since a) Part 1 is sufficient enough for estimating general cognitive abilities [45] and b) differences in performance between children with and without ADHD can emerge with increasing testing time. Such group differences in performance, however, should not be interpreted as differences in IQ values in general but rather can be ascribed to differences in sustaining attention which has been described in ADHD for other speed-limited cognitive tasks [46, 47].

The alertness was tested using the children’s test battery for the assessment of attention (KITAP [48]) submodule”Alertness”, a computer-based vigilance test, in which participants were asked to press a button as fast as possible every time a certain visual stimulus appeared on the screen. In this task, a stimulus (a witch riding on a broom) appeared 30 times on a computer screen (visual angle approx. 14°). The stimulus was presented for a maximum of 5 sec. but disappeared immediately after the response button was pressed. There was a variable inter-stimulus interval of 1.4–4.9 sec. The duration of one session was approximately 2 min. The reaction times of correct responses (reaction times, RT) were recorded in milliseconds (ms) and averaged across four experimental sessions. By averaging the RTs of four different measurements, we expected a reliable, time-stable assessment of the participants´ alertness performance [49].

### Sleep recordings and analyses

In the experimental night polysomnographic (PSG) data were obtained by a portable 33-channel PSG recording device (Somnomedics, Randersacker, Germany). The recordings included electroencephalography (EEG), electrooculogram (EOG), and electromyography (EMG). The EEG was recorded according to the international EEG 10-20-placement system. The EEG electrodes were placed at F3, F4, C3, C4, P3, P4, M1, M2 referenced to Cz during recordings (for later offline analyses, the EEG-signal was referenced to contralateral mastoid electrodes); AFz on the forehead was used as ground. The sampling rate was set to 128 Hz and a band-pass filter (0.2–75 Hz) was applied. The electrodes for the EOG were placed at the lower right and upper left canthi in a diagonal fashion; the sampling rate was 128 Hz, and the band-pass filter was set at 0.2–75 Hz. The EMG was a bipolar submental recording with a sampling rate of 256 Hz and a band-pass filter 0.2–128 Hz.

**Fig 1:**
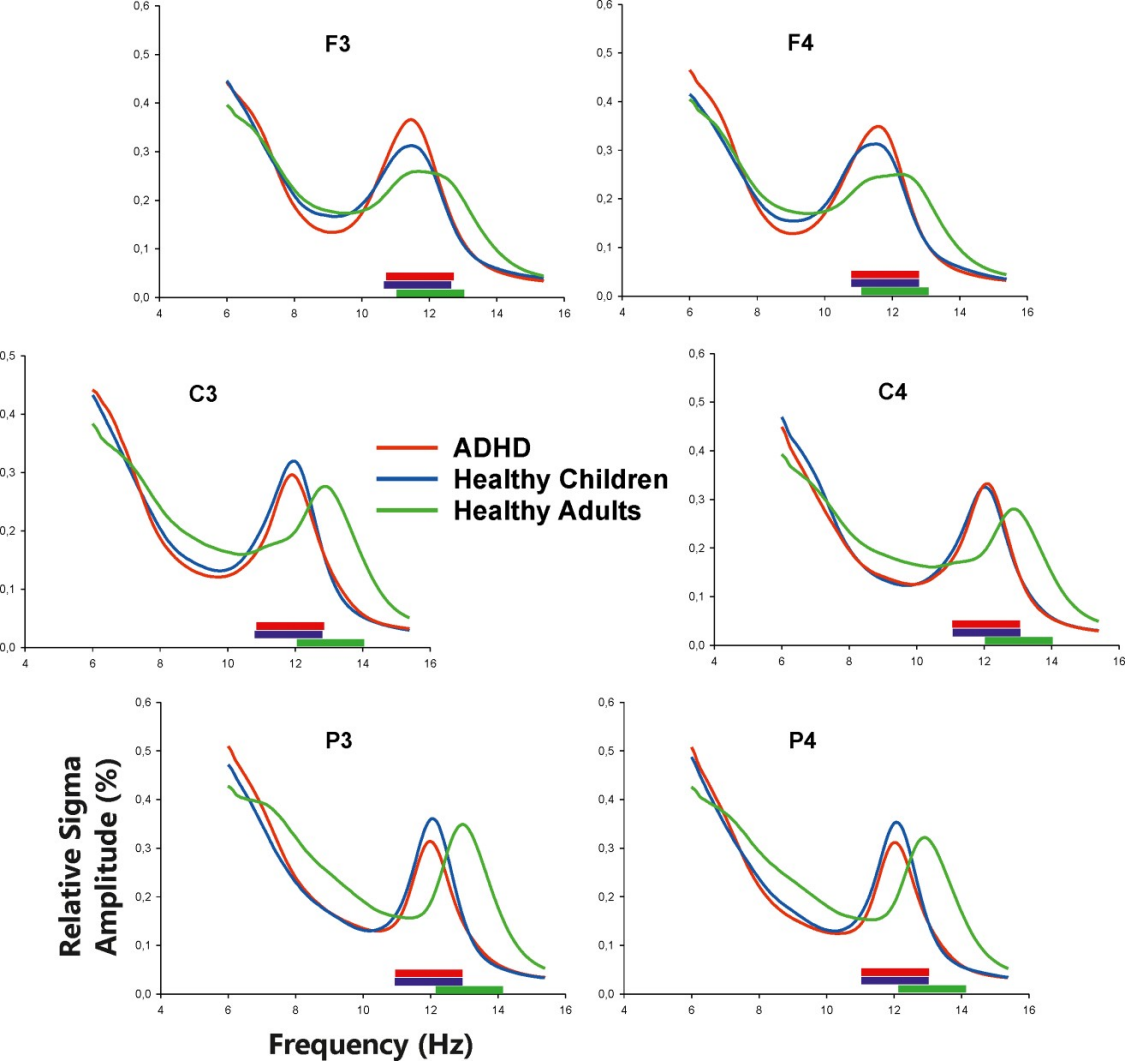
Plots of averages of relative power spectra (depicted from 6 to 15.5Hz) of S2 sleep for children with ADHD (red lines), healthy children (blue lines) and healthy adults (green lines); normalization was applied for 2-25Hz frequency range; colored bars over the x-axis refer to group-specific frequency ranges of power analyses (sigma peak ±1Hz; see also Table 2); ADHD, attention-deficit hyperactivity disorder.

Sleep stages were divided into epochs of 30 sec. and visually scored according to Rechtschaffen & Kales criteria [50] by an experienced examiner. Hereby, the following parameters were calculated: Time in bed (TIB), total sleep time (TST), sleep efficiency (SE), durations of sleep stages S1-4, REM, and non-REM in relation to the TST in percent. For the analysis of sigma activity, the S2 epochs [50] were further divided into segments of 10 sec., and the fast Fourier transform (FFT) algorithm (Hanning window 10%) was used to receive power spectra (in % for relative and in μV^2^ for absolute power values). Brain Vision Analyzer 2 (Brain Products, Gilching, Germany) was used for the power analyses. While the FFT algorithm was performed using a normalization procedure covering the frequency band 2–25 Hz for the relative power analysis, no such normalization procedure was applied for the analyses of absolute power. Individual bad channels (due to irreparable technical problems during ambulant measurements) were replaced by group mean of that specific channel which was the case in only 4% of all data. Then the average power spectrum was calculated for each participant once for relative and once for absolute power. Based on the relative power spectra and according to visual inspection, the frequency of the individual peak of sigma activity was detected within the frequency range of 11–16 Hz for each participant. In a next step, the individual peak frequencies were averaged for each channel and group. These averages were then used to extract the relative and absolute power values around this group- and channel-specific peak frequency with a range of ±1Hz (see also Table 2 and Fig 1). As example: in the case of healthy children for the Position F3 the mean peak frequency was detected at 11.6 Hz; by applying a frequency range of ±1 Hz around this mean the frequency range for the sigma power analysis was set to 10.6–12.6 Hz.

**Table 2:** Sigma activity during S2 sleep.

							ADHD vs. HC	ADHD vs. HA	HC vs. HA
Sigma activity		Mean (SD)	Mean (SD)	Mean (SD)	F	p	p	p	p
Frequency (Hz) of sigma peak	F3	11.7 (0.38)	11.6 (0.44)	12.0 (0.63)	4.2	.020	-	**-**	-
	F4	11.7 (0.40)	11.7 (0.41)	12.0 (0.69)	2.4	.102	-	**-**	**-**
	C3	12.1 (0.22)	12.0 (0.41)	13.0 (0.78)	18.2	**< .001**	.324	**< .001**	**< .001**
	C4	12.2 (0.44)	12.2 (0.30)	13.0 (0.77)	12.5	**< .001**	.976	**.001**	**< .001**
	P3	12.2 (0.21)	12.2 (0.29)	13.2 (0.49)	58.1	**< .001**	.826	**< .001**	**< .001**
	P4	12.2 (0.20)	12.2 (0.30)	13.1 (0.77)	17.9	**< .001**	.757	**< .001**	**< .001**
Relative power (%) +/- 1Hz around peak of sigma activity	F3	0.31 (0.12)	0.28 (0.10)	0.23 (0.08)	3.3	.046	-	-	-
	F4	0.30 (0.12)	0.28 (0.10)	0.23 (0.08)	3.0	.056	-	-	**-**
	C3	0.25 (0.07)	0.26 (0.07)	0.24 (0.09)	0.4	.639	-	-	**-**
	C4	0.26 (0.09)	0.26 (0.10)	0.23 (0.10)	0.4	.665	-	-	**-**
	P3	0.24 (0.08)	0.27 (0.09)	0.28 (0.12)	0.8	.436	-	-	-
	P4	0.24 (0.08)	0.27 (0.12)	0.26 (0.11)	0.4	.668	-	-	-
Absolute power (mV^2^) +/- 1Hz around peak of sigma activity	F3	0.48 (0.25)	0.53 (0.31)	0.12 (0.07)	16.9	**< .001**	.581	**< .001**	**< .001**
	F4	0.48 (0.28)	0.49 (0.31)	0.13 (0.07)	17.8	**< .001**	.921	**< .001**	**< .001**
	C3	0.34 (0.18)	0.34 (0.13)	0.13 (0.07)	16.4	**< .001**	.976	**< .001**	**< .001**
	C4	0.33 (0.17)	0.35 (0.18)	0.14 (0.07)	13.5	**< .001**	.867	**< .001**	**< .001**
	P3	0.19 (0.08)	0.26 (0.14)	0.12 (0.05)	6.1	**.004**	.060	**< .001**	**< .001**
	P4	0.25 (0.16)	0.37 (0.31)	0.14 (0.09)	16.9	**< .001**	.182	.009	**.002**

Note: Frequency (upper row) of sigma activity was detected within the range 11-16Hz of relative power spectra (normalized from 2 to 25Hz); absolute power (lower row) was detected in the frequency band ± 1 Hz around the frequency of the sigma peak (see upper row); ADHD, attention-deficit hyperactivity disorder; HC, healthy children; HA, healthy adults; bold values indicate survival of a Bonferroni-corrected significance level of p < .0083 (0.05/6); see also S4 Table for statistical values (including t-values and CL-95%) of all group-wise comparisons of single means.

### Procedure

Inclusion criteria as well as IQ were assessed during a screening session under laboratory conditions. Alertness performances and PSG were measured in the participants’ home environment. The KITAP alertness test was completed four times: In a PSG condition, alertness was assessed in the evening before sleep and in the morning after sleep (see also [34]). In a second condition, alertness was assessed first in the morning and then in the evening of the same day. Both conditions were conducted at least two weeks apart. For PSG recordings, participants were asked to wear a dummy device with three electrodes the night before real PSG recordings to adapt to the PSG procedure. For real PSG, 17 electrodes were applied between 18:30 and 19:30 p.m. in the children’s groups and between 19:30 and 21:30 p.m. in the adults’ group, depending on the usual individual bedtimes.

### Statistical analyses

SPSS 25.0 (IBM Corp., Armonk, NY, USA) was used to analyze the data. We used univariate analysis of variances (ANOVA) to analyze behavioral and sleep data with the between-subjects factor GROUP (ADHD, healthy children vs. healthy adults). To account for multiple testing with respect to sleep data, we applied a Bonferroni correction. By analyzing six sleep parameters, we set the alpha level at α = .0083 (α = .05/6).

To analyze the sigma peak frequency and sigma power for each sigma parameter, an ANOVA was calculated with the within-factor for POSITION (F3, F4, C3, C4, P3 vs. P4) and the between-subject factor GROUP. If a two-fold interaction reached significance then this interaction was decompensated into six single ANOVAs (one for each position) with the factor GROUP. Here, a Bonferroni-corrected alpha level of α = .0083 (α = .05/6) was used to control for six tests (i.e. six channels) per sigma parameter. In the case of (Bonferroni-corrected) significant main effects, comparisons of single means were performed by t-test for independent samples.

Pearson´s correlation coefficient was used for correlation analysis. Here again, a Bonferroni-corrected alpha level of α = .0083 (α = .05/6) was applied to account for six tests (i.e. six channels) per cognitive task. Only in the case of (alpha adjusted) significant correlation coefficients was the Fisher z-test used to compare the correlation coefficients between the groups.

While only statistical values of those comparisons are depicted in Tables 1–3 that survived the alpha correction, the results of all comparisons can be found in the supplemental material (see S3–S5 Tables).

**Table 3:** Correlations between absolute sigma power and neuropsychological performance.

Cognitive Task	Position	Pearson's correlation coefficient (r)			Comparisons between groups (Fisher's z-transformation)		
		ADHD (n = 17)	HC (n = 16)	HA (n = 23)	ADHD vs. HC	ADHD vs. HA	HC vs. HA
IQ	F3	-.287	.181	.484*	**-**	**-**	-
	F4	.112	.143	.427*	**-**	**-**	-
	C3	.139	.399	.557**	.463	.161	.563
	C4	.072	.218	.584**	.698	.087	.210
	P3	-.487*	.511*	.588**	**.004**	**.001**	.756
	P4	-.216	.307	.671***	.164	**.003**	.164
Alertness (RT)	F3	.085	.529*	-.078	**-**	**-**	-
	F4	-.072	.733**	-.069	**.004**	.497	**.002**
	C3	-.292	.506*	-.144	**-**	**-**	-
	C4	-.266	.614*	-.130	**-**	**-**	-
	P3	.101	.159	-.062	**-**	**-**	-
	P4	.394	.550*	-.474*	**-**	**-**	-

Note: For correlation analyses absolute sigma power values were used; ADHD, attention-deficit hyperactivity disorder; HC, healthy children; HA, healthy adults; bold values indicate survival of a Bonferroni-corrected significance level of p < .0083 (0.05/6); see also S5 Table for statistical values of all group-wise comparisons of correlation coefficients.

*, p < .05 uncorrected

**, p < .005 uncorrected

***, p < .001 uncorrected

## Results

### Neuropsychological performance

All participants had an IQ above 80, and groups did not differ with respect to IQ [F(2,53) = 1.9, p = .156; see Table 1]. The analysis of alertness revealed a main effect for GROUP [F(2,53) = 28.7, p < .001], showing that children with ADHD had prolonged RT compared to HC [t(31) = 2.26, p = .031] and healthy adults [t(38) = 6.92, p < .001). The difference between the RTs of HC and HA was significant as well [t(37) = 7.74, p < .001; for further results see Table 1].

### Sleep parameters

After Bonferroni correction, there were still main effects for GROUP regarding TIB [F(2,52) = 17.0, p < .001], TST [F(2,52) = 14.4, p < .001], S1% [F(2,52) = 6.6, p = .003], and S4% [F(2,52) = 33.2, p < .001], showing that both children´s groups spent more time in bed, slept longer, and had prolonged periods of S1 and S4 than compared to healthy adults (for results of t-tests and further statistics see Table 1). It should be noted that the sleep efficiency was comparable between groups [F(2,52) = 0.2, p = .852]. Most importantly however, both children’s groups did not differ in sleep parameters (see Table 1).

### Sigma activity

With respect to peak frequency of sigma activity, the main effects for GROUP [F(2,53) = 24.7, p < .001] and POSITION [F(5,265 = 45.5, p < .001] as well as the interaction GROUP x POSITION [F(10,265) = 4.2, p < .001] reached significance. A decompensation of this interaction into single (Bonferroni-corrected) ANOVAs revealed that group differences were most pronounced in the central and parietal positions (for descriptive and interference statistics see Table 2). Comparisons of the respective single means revealed that the sigma peak frequency was slower over the central and parietal positions in each child’s group compared to healthy adults (p≤.001). However, children with and without ADHD did not differ in sigma peak frequency (p≥.324).

With respect to the relative power analyses (see Fig 1 and Table 2), there were no significant main effects for POSITION or GROUP (p>.079). Although the interaction GROUP x POSITION reached significance [F(10,265) = 3.6, p < .001], none of the single ANOVAs (with respect to positions) reached Bonferroni-corrected significance (p>.046); therefore, no comparisons of single means were performed.

Regarding absolute sigma power (see also Table 2), there were main effects for GROUP [F(2,53) = 26.6, p < .001] and POSITION [F(5,265 = 15.9, p < .001] as well as a significant interaction GROUP x POSITION [F(10,265) = 4.6, p < .001]. A decompensation of this interaction into single (Bonferroni-corrected) ANOVAs did not yield any additional information since children clearly displayed higher sigma power than adults (p < .004) at all positions. There were no differences between the children´s groups in absolute sigma power at any position (p>.060).

### Correlations between sigma activity and neuropsychological performances

**Fig 2:**
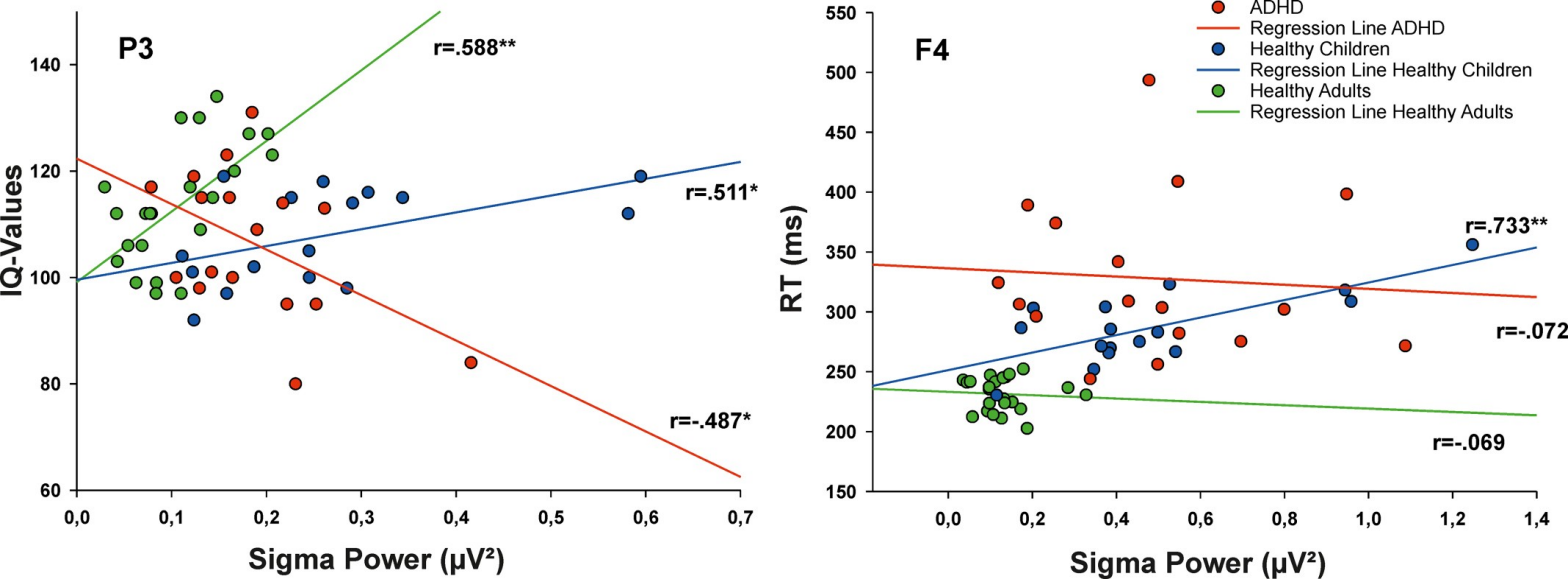
Correlations between the absolute sigma power during S2 (in μV^2^) at the electrode position P3 and IQ-values (left panel) as well as over the electrode position F4 and reaction times (in ms; right panel); ADHD, attention-deficit hyperactivity disorder; *, p < .05 uncorrected; **, p < .005 uncorrected.

After Bonferroni correction in the adults’ group, there were still positive correlations between absolute sigma power and IQ over all central and parietal EEG electrode positions (highest: r = .671, p < .001 over P4, see also Table 3 and Fig 2). In healthy children, there was a positive correlation over P3 as well, which did not survive the alpha-correction, however. Contrary to those results, a significant but uncorrected negative correlation between IQ and absolute sigma power was observed over P3 in the ADHD group (r = -.487, p = .047). Comparisons of correlation coefficients by Fisher´s z-test revealed significant differences between ADHD and healthy adults for positions P3 (p < .001) and P4 (p = .003), as well as between children with and without ADHD for position P3 (p < .004). Regarding relative power values, no correlation survived Bonferroni correction (see S2 Table).

With respect to alertness and absolute power values, there were positive correlations for almost all positions in healthy children. However, the only correlation that survived alpha-correction was between performance and sigma power over F4 (r = .733, p = .001, see also Table 2). Fisher´s z-test confirmed that this correlation coefficient was significantly different to the coefficients obtained from healthy adults (p = .002) and children with ADHD (p = .004). A significant negative correlation between absolute sigma power and alertness over P4 in the healthy adults´ group failed to survive alpha-correction; in the ADHD group, there were no significant correlations between sigma power and alertness at all. With regards to relative power values, no single correlation survived the Bonferroni correction (see S2 Table).

**Fig 3:**
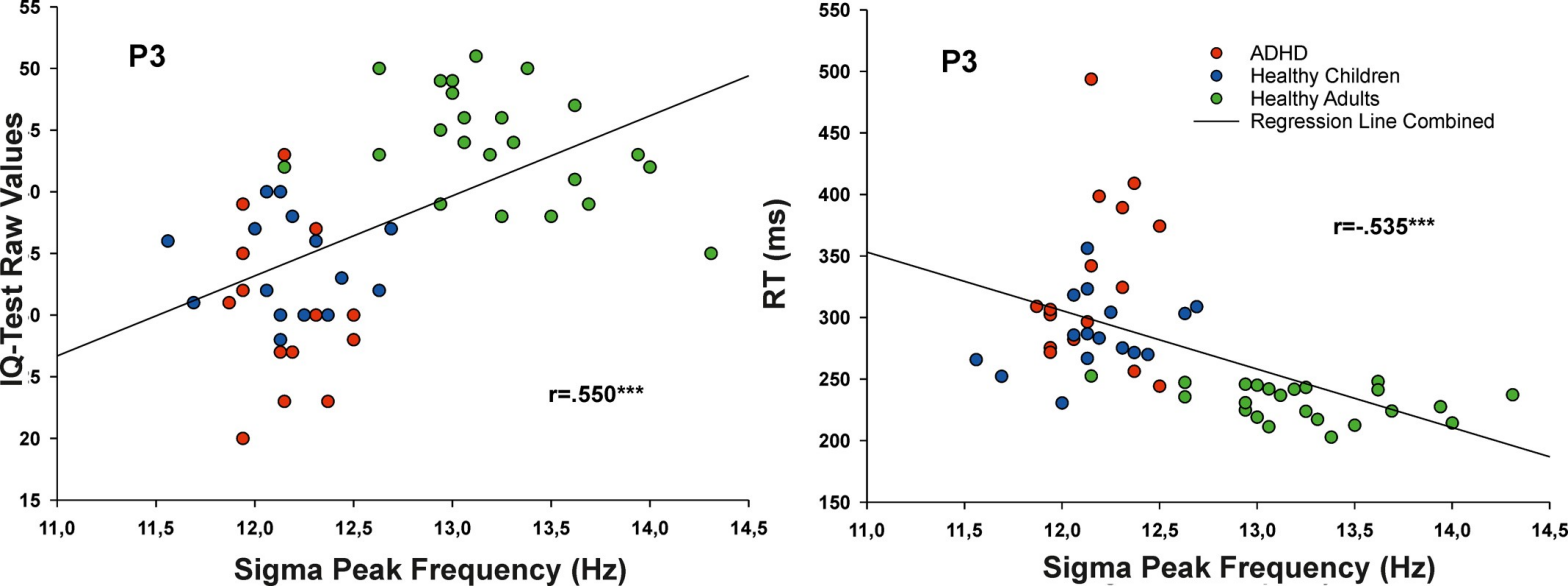
Correlations between the frequency of the peak of relative sigma power during S2 (in Hz) at the electrode position P3 and the IQ-test “CFT-20-R” raw values (left panel) as well as the reaction times (in ms; right panel); ADHD, attention-deficit hyperactivity disorder; ***, p < .005 uncorrected.

To explore relationships between the age-related acceleration in sigma peak frequency and neuropsychological performances, additional Bonferroni-corrected correlations were calculated. There was a significant positive correlation between the sigma peak frequency over P3 and the raw IQ-test values (r = .550, p < .001, see Fig 3). Importantly, these raw data of the IQ-test are not standardized and usually increase with age [45, 51]. Moreover, there was a significant negative correlation for the peak frequency over P3 and reaction times obtained from the alertness task (r = -.535, p < .001). Partial correlations revealed that both correlations were each clearly driven by age [after including age as control variable both above mentioned correlations coefficients were insignificant (p>.3)].

## Discussion

We analyzed correlations between sigma activity and cognitive performance in comparison between children with ADHD and healthy children and adults. While IQ was positively correlated with sigma power in healthy adults, healthy children displayed a positive correlation between sigma power and RT in psychomotor alertness testing. No strong correlations were found in children with ADHD.

Healthy adults displayed faster sigma peak frequency over the central and parietal positions compared to both children’s groups, pointing towards an age-related acceleration of sigma activity. These data are in line with reports [52–54] and can reflect a maturational increase in myelination in thalamo-cortical circuits as an origin of sleep spindles [55]. Taking age-dependent changes of sigma peak frequency into account, sigma power was extracted around the group and channel-specified sigma peak frequency (mean ±1 Hz). As reported before by us and others [12, 13, 34], children showed significantly higher absolute sigma power when than healthy adults. These group differences, however, vanished when comparisons were based on relative power values. Therefore, these data indicate an age-related decrease in oscillatory activity which, however, is not specific to sigma activity (see also [56]). Children with ADHD and healthy children did not show any significant differences in sleep parameters or sigma activity. Thus, we cannot confirm the observations of a selective decrease within the sigma band during sleep stage 2 in ADHD that had been described before in one study [31]. In the same way, no alterations in sigma power have been found in adults with or without ADHD [57]. These divergent findings highlight the yet unmet need to identify the conditions under which sigma power or spindle activity is altered in ADHD.

Regarding absolute sigma power and intellectual performances, we could verify results of earlier studies in the sense of positive correlations between sigma power and IQ in healthy participants [6, 14, 58]. Here, we observed this positive association between sigma power during sleep and intellectual performance in the central/parietal EEG positions in healthy adults; in healthy children, however, such positive associations were only observed on a non-corrected level (highest over the left parietal cortex). These data indicate a maturational process of the function of brain oscillations being first completed over the parietal cortex. This is in line with MRI and post-mortem studies reporting that the parietal cortex is one of the first matured brain areas during pubertal development [59, 60]. Contrary to those findings, the ADHD group did not show such a correlation, and we even found an uncorrected negative correlation between sigma power and IQ over the left parietal cortex. A pronounced unilateral parietal dysfunction in patients with ADHD has been described in several MRI studies [61, 62] [63]. Since ADHD is defined as a neurodevelopmental disorder [19], these data underline a disturbed brain maturation which also obviously affects the relationship between sleep and intellectual performance. In previous studies, we observed that young patients with ADHD did not benefit from oscillatory brain activity during sleep with respect to declarative memory consolidation [33, 34]. Please note that some data of the present study had been published partly before in the latter study. It is important to mention that the absolute power of oscillatory activity during sleep in these studies did not differ between participants with and without ADHD. Therefore, we assumed an insufficient function of oscillatory activity for cognitive performance in ADHD, possibly due to well-described disturbed brain connectivities using imaging techniques [64].

With respect to alertness performance, only healthy adults showed a significant but uncorrected negative correlation over the right parietal cortex. It is important to mention that a negative correlation means that higher sigma power is accompanied by shorter reaction times. In contrast to this and to other studies [58, 65], we found positive correlations between sigma power and alertness performances in healthy children, though the only correlation that survived the alpha-correction was the one over the left frontal position. No such correlations were found in children with ADHD. Doucette and colleagues [65] observed a reverse pattern in younger healthy children (2–5 yrs.): increased slow spindle power (there 10-13Hz) predicted faster processing speed (i.e., shorter reaction time). One possible explanation of these contradicting results might be that the tasks used differ in the level of cognitive and motor demand: While older children were asked to just press a mouse button whenever a stimulus occurred on the screen in our study, younger children were asked to point with a stylus in their hand on a location-invariant stimulus occurring on a computer screen as fast as possible in the study by Doucette and colleagues [65]. Further differences between the study design compared to Doucette and colleagues [65] are the higher sample size and the focus only on male subjects in our study. Geiger and colleagues (2011) [58] did not find any correlations between relative sigma activity and alertness performance in children. In that study the number of healthy children, the age range, the study task, and the calculation of the sigma power were highly comparable to our study. When focusing on relative sigma power, we also did not find any significant correlations between reaction times and sigma activity that survived the alpha correction (see S2 Table). However, since the exact correlation coefficients between sigma and alertness were not reported in the paper of Geiger and colleagues (2011) [58], we cannot say whether their results are at least similar to ours.

Interestingly, when focusing on frontal and central positions, children with ADHD displayed associations between motor performance and sigma power that were more similar to healthy adults than to healthy children. Since especially central EEG potentials can reflect motor-related activity [66], it can be assumed that children with ADHD may display signs of early maturation in the motor cortex. This assumption is supported by the findings of a pre-matured the motor cortex in ADHD [67]. In the same way, it was reported that children with ADHD showed elevated slow wave activity (2–4 Hz) over central positions which was explained by a motor cortex matured early in ADHD [68, 69]. However, the hypothesis that an early matured motor cortex can explain the more adult-like correlations is speculative, especially since this assumption cannot explain why children with ADHD displayed longer reaction times, i.e. deficits in alertness performance as described before [22]. Therefore, further brain imaging data are needed to elucidate whether the adult-like correlation coefficients in ADHD are indeed related to a pre-maturation of the motor cortex. In addition, further studies should employ batteries of cognitive performances covering the full range of cognitive demand. This could confirm our observation that the interplay between cognitive performance, sigma activity, and maturation is also dependent on the complexity of the cognitive task. Finally, the inclusion of medication-naïve patients would be desirable since MPH affects sleep behavior [70, 71], and it is unclear whether a discontinuation of MPH for 48 hours is sufficient to adjust for potential withdrawal or rebound effects.

On an exploratory level we found a positive correlation between the sigma peak frequency over P3 and raw IQ values when combined over all participants. Likewise, there was a negative correlation between sigma power over P3 and alertness reaction times. Please note that raw IQ values as well as reaction times are not age-normed data and, therefore, reflect developmental changes in cognitive performance. In the same way, partial correlations confirmed that both associations are clearly driven by age. These data are in line with a recent study showing that the maturation of sleep spindles is positively correlated with a developmental increase in IQ performance [18]. In that study, however, slow sleep spindle activity over frontal positions showed this association. In contrast to our study, Hahn and colleagues used subtests of multi-dimensional IQ tests (“Wechsler-Scales”) also including verbal IQ. Moreover, they used the age-independent IQ score while we used the age-dependent raw values. It remains unclear whether the topographic differences between the studies are only due to different task demands or due to age-dependent vs. age-independent IQ scores. Nevertheless, we did not only confirm age-related increases in sigma activity and IQ performance and but we also suggest an age-related dynamic in the association between sleep and cognitive performance. In other words: the more advanced the brain maturation, the stronger this association appears. For a better understanding of this dynamic, however, task demands and analysis methods (age-dependent vs. age-independent scores, spindles, vs. sigma; absolute vs. relative power; data-driven vs. a priory defined frequency ranges) also need to be taken into account in further studies.

Taken together, we observed strong positive correlations between sigma power in S2 sleep and IQ only in healthy adults. Sigma power was only strongly associated with motor-related alertness performance in healthy children; children with ADHD resembled the adults’ pattern. These data pinpoint maturational changes in the association between cognitive performance and sigma activity during sleep. Moreover, these data suggest that these associations are relevant for the understanding of healthy and abnormal neurodevelopment.

## Supporting information

S1 Table Overview number of symptoms and diagnoses of ADHD patients. (DOCX)

S2 Table Correlations between relative sigma power and neuropsychological performance. (DOCX)

S3 Table Participants´ characteristics and results, extended. (DOCX)

S4 Table Sigma activity during S2, extended. (DOCX)

S5 Table Correlations between absolute sigma power and neuropsychological performance, extended. (DOCX)

S1 File Sleep questionnaire. (DOCX)
