# Supplementary material for: Associations between cognitive performance and sigma power during sleep in children with attention-deficit/hyperactivity disorder, healthy children, and healthy adults
Source: PLoS One. 2019 Oct 24;14(10):e0224166. doi: 10.1371/journal.pone.0224166 (PMC6812820; doi:10.1371/journal.pone.0224166)
Supplement: S1 Table — (DOCX) [file pone.0224166.s001.docx]

**S1 Table. Overview number of symptoms and diagnoses of ADHD patients**

| Patient No. | No. of inattentive symptoms | No. of hyperactive-impulsive symptoms | Sub-type |
| --- | --- | --- | --- |
| 1 | 9 | 4 | I |
| 2 | 7 | 3 | I |
| 3 | 9 | 8 | C |
| 4 | 7 | 5 | I |
| 5 | 8 | 7 | C |
| 6 | 9 | 6 | C |
| 7 | 7 | 7 | C |
| 8 | 8 | 9 | C |
| 9 | 4 | 9 | H |
| 10 | 8 | 8 | C |
| 11 | 8 | 3 | I |
| 12 | 4 | 8 | H |
| 13 | 7 | 5 | I |
| 14 | 6 | 2 | I |
| 15 | 6 | 0 | I |
| 16 | 7 | 6 | C |
| 17 | 9 | 4 | I |

Note: I, inattentive; H, hyperactive/impulsive; C, combined.
