# Supplementary material for: Associations between cognitive performance and sigma power during sleep in children with attention-deficit/hyperactivity disorder, healthy children, and healthy adults
Source: PLoS One. 2019 Oct 24;14(10):e0224166. doi: 10.1371/journal.pone.0224166 (PMC6812820; doi:10.1371/journal.pone.0224166)
Supplement: S2 Table — (DOCX) [file pone.0224166.s002.docx]

**S2 Table. Correlations between relative sigma power and neuropsychological performance**

| Cognitive Task | Position | Pearson's correlation coefficient (r) | | | Comparisons between groups (Fisher's z-transformation) | | | |
| --- | --- | --- | --- | --- | --- | --- | --- | --- |
|  |  | ADHD (n=17) | HC (n=16) | HA (n=23) | | ADHD vs. HC | ADHD vs. HA | HC vs. HA |
| IQ | F3 | -.130 | .046 | -.056 | | .646 | .830 | .774 |
|  | F4 | -.054 | -.033 | -.022 | | .956 | .927 | .975 |
|  | C3 | -.007 | .277 | .497* | | .449 | .113 | .464 |
|  | C4 | .023 | .177 | .451* | | .686 | .184 | .389 |
|  | P3 | -.205 | .379 | .416* | | .115 | .062 | .902 |
|  | P4 | .090 | .252 | .469* | | .664 | .230 | .481 |
| Alertness (RT) | F3 | .222 | .342 | -.056 | | .735 | .419 | .247 |
|  | F4 | .188 | .484* | -.094 | | .380 | .414 | .081 |
|  | C3 | -.166 | .373 | .037 | | .146 | .557 | .319 |
|  | C4 | -.160 | .272 | .370 | | .253 | .115 | .759 |
|  | P3 | -.121 | .146 | .073 | | .485 | .576 | .836 |
|  | P4 | .090 | .194 | .051 | | .783 | .910 | .683 |

Note: For correlation analyses relative sigma power values were used; ADHD, attention-deficit hyperactivity disorder; HC, healthy children; HA, healthy adults; *, p<.05 uncorrected; no correlation reached significance after Bonferroni correction (p<.0083).
