# Supplementary material for: Associations between cognitive performance and sigma power during sleep in children with attention-deficit/hyperactivity disorder, healthy children, and healthy adults
Source: PLoS One. 2019 Oct 24;14(10):e0224166. doi: 10.1371/journal.pone.0224166 (PMC6812820; doi:10.1371/journal.pone.0224166)
Supplement: S3 Table — (DOCX) [file pone.0224166.s003.docx]

| **S3 Table. Participants´ characteristics and results, extended** | | | | | | | | | | | | | | | | |
| --- | --- | --- | --- | --- | --- | --- | --- | --- | --- | --- | --- | --- | --- | --- | --- | --- |
|  | ADHD (n=17) | HC (n=16) | HA (n=23) |  | ANOVA | |  | t-test | | | | | | | | |
|  |  |  |  |  |  |  |  | ADHD vs. HC | | | ADHD vs. HA | | | HC vs. HA | | |
|  | mean (SD) | mean (SD) | mean (SD) |  | F | p |  | t | p | 95%-CI | t | p | 95%-CI | t | p | 95%-CI |
| Age | 10.7 (1.05) | 10.9 (1.05) | 24.95 (2.86) |  | 335.2 | <.001 |  | 0.6 | .531 | -1.0 – 0.5 | 19.5 | <.001 | -15.7 – -12.8 | 18.7 | <.001 | -15.5 – -12.5 |
| IQ | 106.4 (13.8) | 107.9 (9.0) | 113.2 (11.5) |  | 1.92 | .156 |  | 0.4 | .711 | -9.9 – 6.8 | 1.7 | .097 | -14.9 – 1.3 | 1.5 | .132 | -12.2 – 1.7 |
| RT (ms) | 328.2 (65.4) | 288 (30.6) | 232 (14.0) |  | 28.7 | <.001 |  | 2.3 | .031 | 4.0 – 77.2 | 6.9 | <.001 | 68.4 – 125.0 | 7.7 | <.001 | 41.4 – 70.8 |
| TIB (min) | 601 (64.7) | 580 (59.3) | 492 (64.0) |  | 17.0 | <.001 |  | 0.9 | .368 | -24.9 – 65.2 | 5.3 | <.001 | 67.0 – 150.2 | 4.3 | <.001 | 46.6 – 130.3 |
| TST (min) | 520 (58.1) | 513 (58.6) | 431 (58.4) |  | 14.4 | <.001 |  | 0.3 | .731 | -35.0 – 49.4 | 4.8 | <.001 | 51.2 – 126.6 | 4.2 | <.001 | 42.6 – 121.1 |
| SE | 87 (8.4) | 88.5 (7.2) | 88.1 (7.1) |  | 0.2 | .852 |  | 0.5 | .607 | -7.1 – 4.2 | 0.4 | .678 | -6.0 – 3.9 | 0.2 | .860 | -4.4 – 5.2 |
| S1 (%) | 5.4 (3.3) | 5.2 (1.6) | 8.3 (3.5) |  | 6.6 | .003 |  | 0.2 | .835 | -1.7 – 2.1 | 2.7 | .011 | -5.1 – -0.7 | 3.2 | .003 | -5.1 – -1.1 |
| S2 (%) | 44.2 (6.9) | 48.2 (11.5) | 51.1 (7.5) |  | 3.1 | .052 |  | 1.2 | .238 | -10.8 – 2.8 | 3.0 | .005 | -11.6 – -2.2 | 0.9 | .353 | -9.2 – 3.4 |
| S3 (%) | 12.4 (3.1) | 13.5 (5.7) | 11.1 (4.1) |  | 1.4 | .245 |  | 0.7 | .489 | -4.4 – 2.1 | 1.1 | .280 | -1.1 – 3.7 | 1.5 | .138 | -0.8 – 5.6 |
| S4 (%) | 18.5 (3.5) | 17.2 (3.6) | 8.3 (5.2) |  | 33.2 | <.001 |  | 1.1 | .284 | -1.2 – 4.0 | 7.0 | <.001 | 7.3 – 13.2 | 5.7 | <.001 | 5.7 – 12.0 |
| REM (%) | 19.5 (4.5) | 20.1 (4.7) | 21.2 (4.3) |  | 0.8 | .462 |  | 0.4 | .726 | -3.9 – 2.8 | 1.2 | .226 | -4.6 – 1.1 | 0.8 | .437 | -4.2 – 1.8 |
| Note: ADHD, attention-deficit hyperactivity disorder; HC, healthy children; HA, healthy adults; RT, reaction times; sleep parameters: TIB, time in bed; TST, total sleep time; SE, sleep efficiency; S1-4, sleep stages 1-4; REM, rapid-eye-movement; SD, standard deviation; ANOVA, analysis of variance; CI, confidence interval. | | | | | | | | | | | | | | |  |  |
