# Supplementary material for: Associations between cognitive performance and sigma power during sleep in children with attention-deficit/hyperactivity disorder, healthy children, and healthy adults
Source: PLoS One. 2019 Oct 24;14(10):e0224166. doi: 10.1371/journal.pone.0224166 (PMC6812820; doi:10.1371/journal.pone.0224166)
Supplement: S4 Table — (DOCX) [file pone.0224166.s004.docx]

| **S4 Table. Sigma activity during S2, extended** | | | | | | | | | | | | | | | | | | |  |  |
| --- | --- | --- | --- | --- | --- | --- | --- | --- | --- | --- | --- | --- | --- | --- | --- | --- | --- | --- | --- | --- |
|  |  | ADHD (n=17) | HC (n=16) | HA (n=23) | ANOVA | | t-test | | | | | | | | | | | |  |  |
|  |  |  |  |  |  |  |  | ADHD vs. HC | | | ADHD vs. HA | | | | HC vs. HA | | | | |  |
| Sigma activity |  | Mean (SD) | Mean (SD) | Mean (SD) | F | p |  | t | p | 95%-CL |  | t | p | 95%-CL | | t | p | 95%-CL | | |
| Frequency (Hz) of sigma peak | F3 | 11.7 (0.38) | 11.6 (0.44) | 12.0 (0.63) | 4.2 | .020 |  | 0.8 | .454 | -0.2 – 0.4 |  | 2.0 | .054 | -0.7 – 0 | | 2.5 | .018 | -0.8 – -0.1 | | |
|  | F4 | 11.7 (0.40) | 11.7 (0.41) | 12.0 (0.69) | 2.4 | .102 |  | 0 | .975 | -0.3 – 0.3 |  | 1.7 | .097 | -.07 – 0.1 | | 1.7 | .102 | -0.7 – 0.1 | | |
|  | C3 | 12.1 (0.22) | 12.0 (0.41) | 13.0 (0.78) | 18.2 | <.001 |  | 1.0 | .324 | -.01 – 0.3 |  | 4.4 | <.001 | -1.3 – -0.5 | | 4.6 | <.001 | -1.4 – -0.5 | | |
|  | C4 | 12.2 (0.44) | 12.2 (0.30) | 13.0 (0.77) | 12.5 | <.001 |  | 0 | .976 | -0.3 – 0.3 |  | 3.8 | .001 | -1.2 – -0.4 | | 3.8 | <.001 | -1.2 – -0.4 | | |
|  | P3 | 12.2 (0.21) | 12.2 (0.29) | 13.2 (0.49) | 58.1 | <.001 |  | 0.2 | .826 | -0.2 – 0.2 |  | 8.6 | <.001 | -.13 – -0.8 | | 7.8 | <.001 | -1.3 – -0.8 | | |
|  | P4 | 12.2 (0.20) | 12.2 (0.30) | 13.1 (0.77) | 17.9 | <.001 |  | 0.3 | .757 | -0.2 – 0.2 |  | 4.6 | <.001 | -1.3 – -0.5 | | 4.2 | <.001 | -1.3 – -0.4 | | |
| Relative power (%) +/- 1Hz around peak of sigma activity | F3 | 0.31 (0.12) | 0.28 (0.10) | 0.23 (0.08) | 3.3 | .046 |  | 1.4 | .161 | 0 – 0.2 |  | 1.9 | .060 | 0 – 0.2 | | 0.6 | .535 | -0.1 – 0.1 | | |
|  | F4 | 0.30 (0.12) | 0.28 (0.10) | 0.23 (0.08) | 3.0 | .056 |  | 1 | .305 | -0.1 – 0.2 |  | 2.0 | .056 | 0 – 0-2 | | 1.0 | .324 | 0 – 0.1 | | |
|  | C3 | 0.25 (0.07) | 0.26 (0.07) | 0.24 (0.09) | 0.4 | .639 |  | 0.1 | .942 | -0.1 – 0.1 |  | 1.3 | .203 | 0 – 0.1 | | 1.1 | .261 | 0 – 0.1 | | |
|  | C4 | 0.26 (0.09) | 0.26 (0.10) | 0.23 (0.10) | 0.4 | .665 |  | 0.3 | .739 | -0.2 – 0.1 |  | 1.3 | .217 | 0 – 0.2 | | 1.6 | .126 | 0 – 0.2 | | |
|  | P3 | 0.24 (0.08) | 0.27 (0.09) | 0.28 (0.12) | 0.8 | .436 |  | 0.7 | .463 | -0.2 – 0.1 |  | 0.4 | .708 | -0.1 – 0.1 | | 0.4 | .709 | -0.1 – 0.1 | | |
|  | P4 | 0.24 (0.08) | 0.27 (0.12) | 0.26 (0.11) | 0.4 | .668 |  | 1.0 | .337 | -0.2 – 0.1 |  | 0.1 | .929 | -0.1 – 0.1 | | 1.0 | .338 | -0.1 – 0.2 | | |
| Absolute power (mV^2^) +/- 1Hz around peak of sigma activity | F3 | 0.48 (0.25) | 0.53 (0.31) | 0.12 (0.07) | 16.9 | <.001 |  | 0.6 | .581 | -0.3 – 0.1 |  | 6.5 | <.001 | 0.2 – 0.5 | | 5.9 | <.001 | 0.3 – 0 5 | | |
|  | F4 | 0.48 (0.28) | 0.49 (0.31) | 0.13 (0.07) | 17.8 | <.001 |  | 0.1 | .921 | -0.2 – 0.2 |  | 5.9 | <.001 | 0.2 – 0.5 | | 5.5 | <.001 | 0.2 – 0.5 | | |
|  | C3 | 0.34 (0.18) | 0.34 (0.13) | 0.13 (0.07) | 16.4 | <.001 |  | 0 | .976 | -0.1 – 0.1 |  | 5.2 | <.001 | 0.1 – 0.3 | | 6.4 | <.001 | 0.1 – 0.3 | | |
|  | C4 | 0.33 (0.17) | 0.35 (0.18) | 0.14 (0.07) | 13.5 | <.001 |  | 0.2 | .867 | -0.1 – 0.1 |  | 4.8 | <.001 | 0.1 – 0.3 | | 6.1 | <.001 | 0.1 – 0.3 | | |
|  | P3 | 0.19 (0.08) | 0.26 (0.14) | 0.12 (0.05) | 6.1 | .004 |  | 2.0 | .060 | -0.2 – 0 |  | 3.9 | <.001 | 0 – 0.1 | | 4.9 | <.001 | 0.1 – 0.2 | | |
|  | P4 | 0.25 (0.16) | 0.37 (0.31) | 0.14 (0.09) | 16.9 | <.001 |  | 1.4 | .182 | -0.3 – 0.1 |  | 2.8 | .009 | 0 – 0.2 | | 3.4 | .002 | 0.1 – 0.4 | | |
| Note: Frequency (upper row) of sigma activity was detected within the range 11-16Hz of relative power spectra (normalized from 2 to 25Hz); absolute power (lower row) was detected in the frequency band ± 1 Hz around the frequency of the sigma peak (see upper row); ADHD, attention-deficit hyperactivity disorder; HC, healthy children; HA, healthy adults; CI, confidence interval. | | | | | | | | | | | | | | | | | | | | |
