# Supplementary material for: Associations between cognitive performance and sigma power during sleep in children with attention-deficit/hyperactivity disorder, healthy children, and healthy adults
Source: PLoS One. 2019 Oct 24;14(10):e0224166. doi: 10.1371/journal.pone.0224166 (PMC6812820; doi:10.1371/journal.pone.0224166)
Supplement: S5 Table — (DOCX) [file pone.0224166.s005.docx]

**S5 Table. Correlations between absolute sigma power and neuropsychological performance, extended**

| Cognitive Task | Position | Pearson's correlation coefficient (r) | | | Comparisons between groups (Fisher's z-transformation) | | | |
| --- | --- | --- | --- | --- | --- | --- | --- | --- |
|  |  | ADHD (n=17) | HC (n=16) | HA (n=23) | | ADHD vs. HC | ADHD vs. HA | HC vs. HA |
| IQ | F3 | -.287 | .181 | .484* | | .214 | .018 | .333 |
|  | F4 | .112 | .143 | .427* | | .935 | .324 | .381 |
|  | C3 | .139 | .399 | .557** | | .463 | .161 | .563 |
|  | C4 | .072 | .218 | .584** | | .698 | .087 | .210 |
|  | P3 | -.487* | .511* | .588** | | .004 | .001 | .756 |
|  | P4 | -.216 | .307 | .671*** | | .164 | .003 | .164 |
| Alertness (RT) | F3 | .085 | .529* | -.078 | | .191 | .639 | .061 |
|  | F4 | -.072 | .733** | -.069 | | .009 | .993 | .005 |
|  | C3 | -.292 | .506* | -.144 | | .026 | .655 | .049 |
|  | C4 | -.266 | .614* | -.130 | | .010 | .684 | .018 |
|  | P3 | .101 | .159 | -.062 | | .878 | .639 | .532 |
|  | P4 | .394 | .550* | -.474* | | .600 | .007 | .001 |

Note: For correlation analyses absolute sigma power values were used; ADHD, attention-deficit hyperactivity disorder; HC, healthy children; HA, healthy adults; *, p<.05 uncorrected; **, p<.005 uncorrected, ***, p<.001 uncorrected.
