# Supplementary material for: Associations between cognitive performance and sigma power during sleep in children with attention-deficit/hyperactivity disorder, healthy children, and healthy adults
Source: PLoS One. 2019 Oct 24;14(10):e0224166. doi: 10.1371/journal.pone.0224166 (PMC6812820; doi:10.1371/journal.pone.0224166)
Supplement: S1 File — (DOCX) [file pone.0224166.s006.docx]

**S1 File. Sleep questionnaire**

| Date: |  |
| --- | --- |
| Code |  |

**Sleep questionnaire for parents**

The following questions will help us to get a general survey of the health status and the sleep behavior of your child. Please fill out this questionnaire by ticking the boxes and providing the information in the appropriate places. If there are any questions or uncertainties, please contact the experimenter.

**1) Current health status**

Weight: _______ in kg Height: _______ in cm

|  | *please tick the relevant boxes* | if yes… |
| --- | --- | --- |
| Infection of the upper respiratory tract | no □ yes □ | since: |
| Vaccination (less than 7 days ago) | no □ yes □ | which: |
| Other acute illness | no □ yes □ | which: since: |
| Medication | no □ yes □ | which: *(dose)* |

**2) Did or does your child suffer from any of these diseases?**

|  | *please tick the relevant boxes* | if yes, in which period: |
| --- | --- | --- |
| bronchial asthma | no □ yes □ |  |
| frequent cough | no □ yes □ |  |
| obesity | no □ yes □ |  |
| enlarged tonsils | no □ yes □ |  |
| kissing tonsils | no □ yes □ |  |
| enlarged adenoids/polyps | no □ yes □ |  |
| frequent stomach ache | no □ yes □ |  |
| choanal atresia or stenosis | no □ yes □ |  |
| frequent aspiration | no □ yes □ |  |
| frequent fever (of unknown origin) | no □ yes □ |  |
| chronic respiratory infections | no □ yes □ |  |
| large tongue or incomplete closure of the mouth | no □ yes □ |  |
| funnel chest or chicken chest (chest malformation) | no □ yes □ |  |
| scoliosis | no □ yes □ |  |
| cleft palate | no □ yes □ |  |
| (congenital) heart defect | no □ yes □ |  |
| other acute or chronic diseases, please describe: | no □ yes □ |  |

**3) Symptoms**

| Your child… | *please tick the relevant boxes* |
| --- | --- |
| … is hard to wakeup in the morning and awakens slowly (“not a morning person”) | no □ rarely □ sometimes □ often □ always □ |
| … suffers from dryness of the mouth in the morning | no □ rarely □ sometimes □ often □ always □ |
| … suffers from headache in the morning | no □ rarely □ sometimes □ often □ always □ |
| … has “sticky” eyes in the morning | no □ rarely □ sometimes □ often □ always □ |
| … is tired during the day | no □ rarely □ sometimes □ often □ always □ |
| … falls asleep during the day apart from midday nap | no □ rarely □ sometimes □ often □ always □ |
| … wakes up at night | no □ rarely □ sometimes □ often □ always □ |
| … sweats while sleeping | no □ rarely □ sometimes □ often □ always □ |
| … sleeps restlessly | no □ rarely □ sometimes □ often □ always □ |
| … snores at night | no □ rarely □ sometimes □ often □ always □ |
| … goes to the toilet at night or suffers from enuresis after the third year of life | no □ rarely □ sometimes □ often □ always □ |

**4) Sleep behavior of your child**

| Average sleep duration after meals  after breakfast: …… hours after lunch:…… hours after dinner:…… hours |
| --- |
| Preferred posture during sleep: supine position □ prone position □ side position □ |
| Total sleep time (daytime): |
| Total sleep time (nighttime): |
| How often does your child wake up at night? |
| Does your child settle down well before bedtime? |
| If your child lies in bed, how long does it take for him/her to fall asleep? |
| other comments: |

**5) Breathing during sleep**

| While sleeping your child has … | *please tick the relevant boxes* |
| --- | --- |
| … irregular breathing? | no □ rarely □ sometimes □ often □ always □ |
| … snoring breathing? | no □ rarely □ sometimes □ often □ always □ |
| … pauses in breathing? | no □ rarely □ sometimes □ often □ always □ |
| … pauses in breathing with symptoms? | no □ rarely □ sometimes □ often □ always □ |
| Maximum duration of pause in breathing: | 5 sec □ 10 sec □ 15 sec □ 20 sec □ > 20 sec □ |
| Other abnormalities in breathing? | no □ which? |
| Does your child need artificial respiration at night? | which? pressure: |

**Thank you!**
